# Environmental factors and river network position allow prediction of benthic community assemblies: A model of nematode metacommunities

**DOI:** 10.1038/s41598-019-51245-2

**Published:** 2019-10-11

**Authors:** Birgit Gansfort, Walter Traunspurger

**Affiliations:** Animal Ecology, Bielefeld University, Konsequenz 45, 33615 Bielefeld, Germany

**Keywords:** Community ecology, Ecological modelling

## Abstract

The field of metacommunity studies is growing rapidly, including recent applications to river networks. Most of these studies have targeted a single river network but whether their findings are relevant to other river systems is unknown. This study investigated the influence of environmental, spatial and temporal parameters on the community structure of nematodes in the river networks of the Elbe and Rhine. We asked whether the variance in community structure was better explained by spatial variables representing the watercourse than by overland distances. After determining the patterns in the Elbe river network, we tested whether they also explained the Rhine data. The Elbe data were evaluated using a boosted regression tree analysis. The predictive ability of the model was then assessed using the Rhine data. In addition to strong temporal dynamics, environmental factors were more important than spatial factors in structuring riverine nematode communities. Community structure was more strongly influenced by watercourse than by Euclidean distances. Application of the model’s predictions to the Rhine data correlated significantly with field observations. Our model shows that the consequences of changes in environmental factors or habitat connectivity for aquatic communities across different river networks are quantifiable.

## Introduction

Given that habitat fragmentation and environmental change are progressive, a theoretical framework is needed that allows prediction of the consequences for ecological communities. Expanding studies beyond a local spatial scale to a regional view can shed light on how local community dynamics are affected by the regional species pool^1^. Over the last decade, this metacommunity concept has become a powerful tool for determining the relative importance of spatial (i.e. regional, dispersal-related) and environmental (local, niche-based) processes in structuring communities^2^. If dispersal rates are not a limiting factor at the spatial scale of investigation, then species will track along environmental gradients, with their respective populations structured according to their preferred environmental conditions (i.e., species sorting)^1^. If dispersal is limited, such that species cannot reach potentially suitable habitats (dispersal limitation), or if the rate of dispersal is very high, such that communities become homogenized as member species are distributed into neighboring habitats irrespective of their suitability (i.e. mass effects), then community assembly will be related to spatial factors^2^.

The degree of site connectivity along a dendritically structured river network strongly influences metacommunity structure^3^. However, quantifying the amount of connectivity between sites remains challenging^3^, as the dispersal rates within different ecosystems and for entire species assemblages are not easily formulated^4^. An alternative approach is to use spatial variables as proxies for dispersal intensity, as shorter physical distances between sites imply stronger connectivity between communities via dispersal.

The choice of a distance measurement as the spatial variable depends on the dispersal mode of the studied organism^5^. Although the Euclidean distance, defined as the straight line between two sites, is often used^6^, it reflects only the dispersal routes of an organism over land and is not appropriate for the dispersal of aquatic organisms along a watercourse, which instead requires measurement of the distances between sites along that body of water. The importance of these two dispersal proxies for community assemblies widely varied according to the studied organismal group, thus showing that different taxa respond differently to isolation effects in stream networks^7^.

In addition to differences arising for reasons of biology and landscape setting, some of the discrepant results in those studies may have been due to choices made by the researchers in analyzing the data^3^. For example, whereas presence/absence (p/a) data were used by Heino *et al*.^8,9^, abundance data were analyzed in the studies of Landeiro *et al*.^10^. However, different processes might drive the presence vs. abundance of species, requiring that both be taken into account^11^.

Interestingly, studies investigating the same organismal group and using similar analytical techniques have reached different conclusions concerning the impact of spatial and environmental variable groups (e.g. for macroinvertebrates^9,12^ or for fishes^7,9^). Therefore, the question arises whether general drivers of aquatic communities can be identified and whether they are applicable to different river systems. Alternatively, there may be very specific mechanisms that occur in each river system (hereinafter called river network identity) such that general predictions are impossible. Distinguishing between these two possibilities requires the application of a model that allows predictions of community similarities across river networks. This type of model should also allow a simultaneous determination of the drivers of aquatic communities and a quantification of the consequences of changes in both the environment and connectivity for the species assemblies in a particular river habitat. The two river systems investigated in this study were those of the Elbe and Rhine, which are among the ten longest rivers in Europe. The river systems are not connected by a waterway, but the courses of both of the respective rivers traverse Germany from south to north, such that the climatic conditions and the species pools occurring in their habitats are similar.

Metacommunities of river networks have been studied frequently with regard to a wide range of organismal groups, from bacteria to fishes. By contrast, within the broadly diverse and highly abundant meiobenthos, only ostracods have been studied in this context^13,14^. Since, among aquatic animals, the meiobenthos are of intermediate size (most are 1–2 mm long) and mobility (benthic, passive dispersers), they are useful in studies of the role of dispersal capacity in metacommunity processes.

Among meiobenthic freshwater taxa, nematodes are the most abundant and diverse^15^. As such, they are an essential trophic link between unicellular (e.g., bacteria) and larger organisms (e.g., macroinvertebrates and fishes)^16,17^. Moreover, while nematodes can move actively over short distances (mm–cm), their long-distance distribution depends on passive dispersal. Thus, along a watercourse they can be drifted within the body of water^18^ whereas overland dispersal depends on vectors such as wind, rain, or other animals^19,20^.

As ostracods are so far the only meiobenthic organisms in streams to have been studied within the metacommunity context, we base our expectations on the results of those two studies^13,14^. Both showed that spatial and environmental variables were related to the ostracod metacommunity whereby spatial factors, especially watercourse distances, dominated. Therefore, we suggest that nematode communities will be primarily structured by spatial factors, with environmental factors playing a significant but still secondary role.

In this study, we investigated (1) the relative influence of spatial vs. environmental factors in structuring the nematode metacommunity in the riverine network of the Elbe river with respect to pure species assemblages (p/a data) and community abundance compositions. Specifically, we asked whether the variance in community structure was better explained by spatial variables derived from the watercourse than by overland distances. We hypothesized that (H1.1) nematode communities are primarily structured by spatial factors and that (H1.2), among the spatial factors, distances along the watercourse are a more important determinant of community similarities than are overland distances. (2) We then examined the extent to which the patterns observed in the Elbe river network could be transferred to distinct river systems, by testing the overlap between predictions based on a model developed using the Elbe data and real observations from the Rhine river network. We hypothesized (H2) that a model based on environmental, spatial and temporal dynamics would be able to predict the community similarities of nematodes in a distinct but geographically proximate river system.

## Material and Methods

### Sampling sites

**Figure 1:**
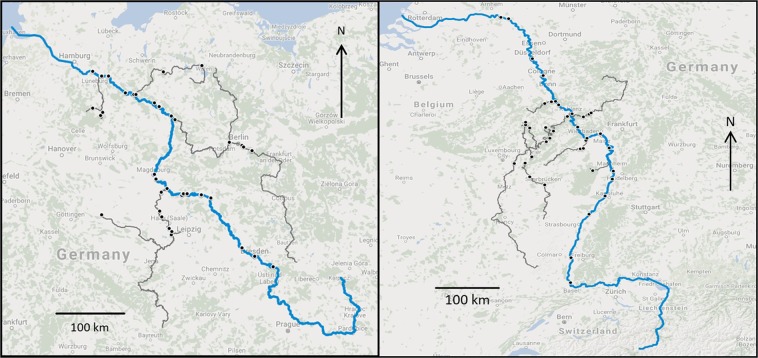
Map of the sampling sites (black points) within the Elbe (left) and Rhine (right) river systems. The blue line indicates the Elbe and Rhine rivers, and the gray lines the tributaries.

Between 2000 and 2013, the Federal Institute of Hydrology (BfG) collected 115 samples within the Elbe and 59 samples within the Rhine river systems. The 115 Elbe samples were obtained from 47 sampling sites (Fig. 1 left), with 25 sites sampled only once and 22 repeatedly (2–10 samples). The 59 Rhine samples were obtained from 42 sampling sites (Fig. 1, right), with only 2 sampling sites sampled repeatedly (7 and 12 samples). The sampling sites of Elbe river system were located in 10 rivers, with 39 samples taken from tributaries and 76 along the Elbe itself. In the Rhine river system, samples were taken from 7 rivers, with 24 from tributaries and 35 from along the Rhine itself.

### Sample processing

Samples for nematode analysis were collected and processed as described in earlier studies^21,22^, specifically nematode extraction was conducted by density gradient centrifugation in colloidal silica (LUDOX)^23^. Three subsamples from the uppermost 2–3 cm of sediment were collected and pooled. From each of these pooled samples the first 150 nematodes (if present) were identified to the species level (Leica Dialux microscopy observations, 1250× magnification), mainly according to the criteria of Andrássy^24–26^ and Loof^27,28^. In total, 12,370 individuals were identified in the Elbe samples and 8,520 in the Rhine samples. The physico-chemical parameters of the sediment were determined in additional grab samples (top layer 0–10 cm) obtained from the same sites as the nematode samples, with at least 10 subsamples combined to overcome the effects of local variability (for details, see Heininger *et al*.^21^). All sampling was conducted using the same sampling protocol.

Sampling and the subsequent analysis of sediment properties and contamination (heavy metals and organic chemicals) were carried out according to Heininger *et al*.^21^. The measured sediment characteristics included (for summary see Table 1, raw data are given as Supplementary Information Table S1): grain size distribution (four categories: F1 > 2000, F2 60–2000, F3 20–60, and F4 < 20 µm), total organic carbon (TOC) and the elements N and S (Elbe: 15 missing values each; Rhine: 10 missing values each); Al, Ca, Fe, Li, Mg, Mn, and P (Elbe: 42 missing values each; Rhine: 23 missing Al values, 30 missing Li values, all other elements: 28 missing values). The following contaminants were analyzed in the sediments: metals [Cd, Cr, Cu, Hg (Elbe: 2 missing values), Ni, Pb, and Zn]; As; seven PCBs [PCB 28 (one missing value), 52, 101, 118, 138, 153, 180]; 16 PAHs (according to the priority pollutant list of the US-Environmental Protection Agency(EPA): acenaphthene, acenaphthylene; anthracene; benz[a]anthracene; benzo[a]pyrene; benzo[b]fluoranthene; benzo[g,h,i]perylene; benzo[k]fluoranthene; chrysene; dibenz[a,h]anthracene; fluoranthene; fluorene; indeno[1,2,3-cd]pyrene; naphthalene (3 missing values); phenanthrene; pyrene]; DDTs (pp′-DDT, pp′-DDD, pp′-DDE; HCB); and mineral oils. The missing values were those not available from the different authorities responsible for measuring the chemical parameters during the long sampling period. However, our method of analysis was able to adequately handle missing values^29^. In addition, there were relatively few missing values, with the exception of some of the Rhine samples (Fe, Li, Mg, Mn, P). While missing values could have led to an underestimation of model performance for the Rhine data, it was not expected to influence either model building or assessments of the influence of predictors within the model.

**Table 1:** Summary statistics of the physical, ecotoxicological, and spatial variables and of the elements from 115 samples from the Elbe river system and 59 samples from the Rhine river system.

Variable	Unit	Mean (SD)	Min	Max
**Elbe**				
Physical				
Grain-size fraction F1	%	2.4 (6.0)	0	51.8
Grain-size fraction F2	%	40.4 (26.1)	1.1	99.7
Grain-size fraction F3	%	16.6 (10.2)	0.3	47.2
Grain-size fraction F4	%	39.5 (23.0)	0	87.4
River km	km	520.2 (255.4)	2.9	950.4
Element				
Aluminum (Al)	%	3.3 (1.0)	0.7	6.4
Calcium (Ca)	%			
Ferric (Fe)	%	4.8 (1.6)	1.0	9.3
Lithium (Li)	mg/kg			
Magnesium (Mg)	%	0.6 (0.2)	0.09	1.2
Manganese (Mn)	mg/kg	1,843.1 (1169.8)	444.0	6,240.3
Nitrogen (N)	g/kg	3.2 (3.2)	0	19.0
Phosphorus (P)	g/kg	3.7 (1.8)	0.9	12.3
Sulphur (S)	g/kg	3.1 (3.4)	0	15.0
Total organic carbon (TOC)	g/kg	38.7 (30.5)	0.1	155.2
Ecotoxicological				
Mean PEC-Q_met_		2.3 (1.9)	0.02	15.6
Mean PEC-Q_org_		2.4 (3.2)	0.01	23.5
Spatial				
Geographical PCoA vector 1		5,745.5 (101,193.2)	−168,133.3	208,930.9
Geographical PCoA vector 2		−3,162.4 (40,087.5)	−130,493.3	111,076.2
Dendritic PCoA vector 1		32.5 (170.1)	−244.6	343.8
Dendritic PCoA vector 2		−12.4 (75.2)	−175.7	250.1
Altitude	m	52.3 (43.7)	1	377
**Rhine**				
Physical				
Grain-size fraction F1	%	5.8 (13.5)	0.0	58.5
Grain-size fraction F2	%	30.7 (25.8)	0.4	97.3
Grain-size fraction F3	%	14.7 (8.5)	0.2	37.0
Grain-size fraction F4	%	46.9 (25.9)	0.2	93.9
River km	km	543.3 (367.8)	2.5	1062.8
Element				
Aluminum (Al)	%	5.7 (4.0)	2.5	18.0
Calcium (Ca)	%	3.4 (3.1)	0.4	12.1
Ferric (Fe)	%	3.8 (0.6)	2.4	4.8
Lithium (Li)	mg/kg	61.7 (19.2)	33.2	105.9
Magnesium (Mg)	%	1.0 (0.3)	0.5	1.6
Manganese (Mn)	mg/kg	1142.9 (472.8)	565.3	3001.6
Nitrogen (N)	g/kg	1.4 (1.5)	0.1	5.6
Phosphorus (P)	g/kg	1.8 (0.5)	1.0	3.1
Sulphur (S)	g/kg	0.8 (1.0)	0.0	4.9
Total organic carbon (TOC)	g/kg	16.6 (14.9)	0.2	46.0
Ecotoxicological				
Mean PEC-Q_met_		0.8 (0.6)	0.04	4.8
Mean PEC-Q_org_		0.5 (0.3)	0.01	1.5
Spatial				
Geographical PCoA vector 1		−2,8491.0 (105,633.2)	−223,190.5	260,480.1
Geographical PCoA vector 2		2,360.4 (38,177.9)	−82,108.6	66,033.2
Dendritic PCoA vector 1		−3.0 (140.2)	−274.1	431.4
Dendritic PCoA vector 2		−40.6 (149.8)	−355.7	152.4
Altitude	m	110.4 (100.6)	8.0	460.0

### Variable selection

#### Spatial variables

The location (latitude, longitude, altitude) of the sampling sites was documented by the BfG during sampling and the shortest distance from each sampling site to the river source along the river (river km) was measured. The two categories of spatial variables used in the analysis consisted of: geographical position (Euclidean distance, corrected for the Earth’s curvature) and dendritic position (watercourse distance). The geographical position was characterized using the first two vectors from a principal coordinates analysis (PCoA) of the distance matrix generated by determining the geodesic distance between all sites (calculated using the R package *geosphere*^29^). These two vectors constituted >99.9% of the eigenvalues for the distance matrix (for the Elbe and Rhine sampling sites, respectively).

For the dendritic position of the sites, a second distance matrix measuring the distances of each sampling site to all other sampling sites along the watercourse was created. A PCoA was performed for this dendritic distance matrix as well and the first two vectors used as variables for the dendritic position of the sites. The two vectors constituted 78% and 92% of the eigenvalues for the distance matrices of the Elbe and Rhine river systems, respectively. A comparison of the PCoA plots for the Elbe and Rhine data is provided in the Supplementary Information (Fig. S1).

Both PCoAs were conducted using the w*cmdscale* function in the R package *vegan*^30^.

### Calculation of a sediment-quality-based index

The toxic potential of the sediment was determined using an index based on sediment quality criteria defined for each of the measured contaminants. Threshold sediment concentrations of the contaminants, above which toxic effects on benthic macroinvertebrates are expected (i.e., probable effect concentrations, PECs), were taken from De Deckere *et al*.^31^. The PEC quotient (PEC-Q) was calculated for each chemical, with the mean PEC-Q of eight metals (mean PEC-Q_met_) and 26 organic chemicals (mean PEC-Q_org_) serving as a good approximation of the combined effects of the multiple contaminants in the sediment^32^.

### Nematode community

The dataset for the Elbe river system comprised 115 samples and that for the Rhine 59 samples. The similarities of the communities were estimated using a non-metric multidimensional scaling (NMDS) procedure, calculated using the Bray-Curtis similarity index, and applied to the abundance and to the p /a data of the Elbe and Rhine samples. For each dataset, three NMDS axes were included, resulting in a stress value of 0.19 for both. The Inclusion of more than three axes appeared to be meaningless, as the resulting reduction in stress was insignificant. NMDS was calculated using the *metaMDS* function of the *vegan* package^30^ in the *R* environment.

### Boosted regression tree (BRT) analysis

#### Calculation of a BRT model using Elbe data

A boosted regression tree (BRT) analysis was used to identify the variables potentially responsible for the structure of the nematode community. A comprehensive introduction to the application of BRT models in ecological datasets is given in Elith *et al*.^33^. BRT models combine a number of decision trees to yield one large model. The optimal *number of trees* was defined using *k*-fold cross-validation (*k* = 10) to avoid overfitting. In each step of BRT model development, a certain proportion of the dataset is drawn at random. The parameter that specifies the size of this proportion is the *bag fraction* (*bf*). In our study, the *bf* was set to 0.75. The *learning rate* (*lr*) defines the degree to which each new tree contributes to the whole model and in this study was set to *lr* = 0.001, such that in each model at least 1000 trees were reached, as recommended by Elith *et al*.^33^. The *tree complexity* (*tc*) reflects the interaction depth and was set to *tc* = 2, as recommended for relatively small data sets^33^.

The relative influence (RI) of each predictor was calculated in the single decision trees using the sum of squared improvements at all splits determined by the predictor. This relative influence was then averaged over the >1000 trees included in the whole BRT model and scaled between 0 and 100^34^. Therefore, the RI values were not equivalent to the percentages of the explained variance, as is the case in other statistical methods often used in metacommunity assessments. In addition, a *p* value indicating the significance of the model’s coefficients vs. conventional regression models could not be defined using BRT models^33^. However, the higher the RI value of a predictor, the greater its contribution to the sorting of the dataset by the whole BRT model. Therefore, in our analysis, the RIs were proportional to the influence of these variables on nematode community structure.

For cross-validation and calculation of the model, the *gbm.step* function of the *dismo* package^35^ in the *R* environment was applied.

BRT models were calculated using all of the predictors listed in Table 1 and two temporal variables (Year: 2000–2013; Season: Spring, Summer, Autumn, Winter) and each of the three axes of the NMDS of nematode abundance and p/a data as response variables (in total, 6 models calculated). Grain-size fraction F4 was excluded from the predictor set in the models because it was already determined by the other three grain-size fractions (in total: 100%).

For a better interpretability of the results we summarized the RI for the three NMDS axes into a single RI (for p/a and abundance data respectively) by calculating a weighted average. Weighting was based on the relative stress reduction resulting from the inclusion of one additional axis into the NMDS model (explained in Supplementary Information Text). We are aware that NMDS axis 2 in a two-dimensional plot is not the same as NMDS axis 2 in a three-dimensional plot. However, the two axes are extremely similar (the correlation coefficients were >0.94) and the better interpretability offered by the former justifies its use.

### Using the Elbe model to predict the Rhine data

The performance of models obtained from the Elbe data were tested using the Rhine data. Therefore, predictions for the Rhine communities were obtained using the model constructed from the Elbe data, by supplying it with the abiotic data describing the Rhine samples. The predicted community similarities were than compared to the real community similarities of the Rhine samples. Predictions were made using the *predict.gbm* function of the *gbm* package^36^.

The predictive performance of the models on the Rhine river system was evaluated using evaluation statistics according to Potts and Elith^37^:

1. Pearson’s correlation coefficient (*r*) was used to test the relative similarity of the observed and predicted values; a perfect correlation (r = 1) does not imply that the predictions were exact, but that they were either correct or biased in a consistent direction.
2. Spearman’s rank correlation (*ρ*) provided an indication of how the degree of agreement between the ranks of observed and predicted values.

### Ethical approval

All applicable institutional and/or national guidelines for the care and use of animals were followed.

## Results

### Nematode community

**Figure 2:**
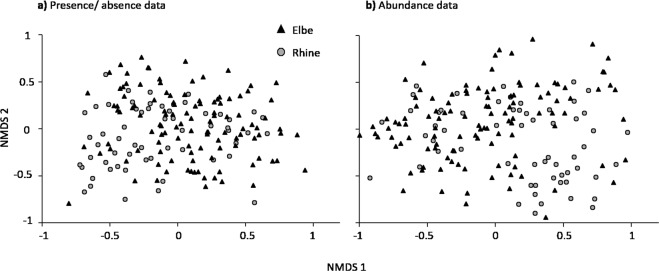
NMDS plots of nematode presence/absence (**a**) and abundance (**b**) data from 115 Elbe (triangles) and 59 Rhine (circles) samples.

The 115 samples comprising the Elbe dataset (Supplementary Information Table S2) and the 59 samples comprising the Rhine dataset (Table S2) contained 245 and 231 nematode species, respectively. A detailed list of nematode abundances in each sample is given as supplementary information (Table S1). From 314 species that were identified, the 10 most common species are listed in Table 2. Seven of these species occurred in both river systems (Table 2). *Eumonhystera filiformis* and *Tobrilus gracilis* predominated in the Elbe and Rhine river systems, occurring in >85% of the samples, whereas 69 species occurred solely in the Rhine samples and 83 solely in the Elbe samples. The NMDS of abundances and the p/a data divided the Elbe and Rhine samples mostly along the first axis but with considerable overlap of the respective communities (Fig. 2).

**Table 2:** Ten most common nematode species of the 115 Elbe and 59 Rhine samples; numbers in parenthesis means species that are not within the ten most common of the respective river system.

Species	Number of presences in		
	Total	Rhine	Elbe
*Eumonhystera filiformis* (Bastian, 1865)	167	54	113
*Tobrilus gracilis* Bastian, 1865	149	52	97
*Monhystera paludicola* de Man, 1880	134	45	89
*Dorylaimus stagnalis* Dujardin, 1845	113	45	68
*Eumonhystera pseudobulbosa* (Daday, 1896)	111	40	71
*Monhystera stagnalis* Bastian, 1865	100	41	59
*Brevitobrilus stefanskii* (Micoletzky, 1925)	93	(23)	70
*Eumonhystera vulgaris* (de Man, 1880)	81	35	46
*Daptonema dubium* Bütschli, 1873	78	35	(43)
*Eumonhystera longicaudatula* (Gerlach & Riemann, 1973)	76	32	(44)
*Tripyla glomerans* Bastian, 1865	72	(16)	56
*Filenchus vulgaris* (Brzeski, 1963)	69	35	(34)
*Chromadorita leuckarti (de Man, 1876)*	61	(15)	46

### Model output

In most cases the performance of the BRT models was satisfactory, as indicated by the low total deviance and high correlation coefficients within the cross-validation process (Table S3). Regarding the first NMDS axis there was a drop in the correlation between the training and cross-validation data, suggesting the occurrence of overfitting. This effect might have been due to the limited amount of data and thus the small proportion available for model cross-validation. However, it did not appear in the other models.

**Figure 3:**
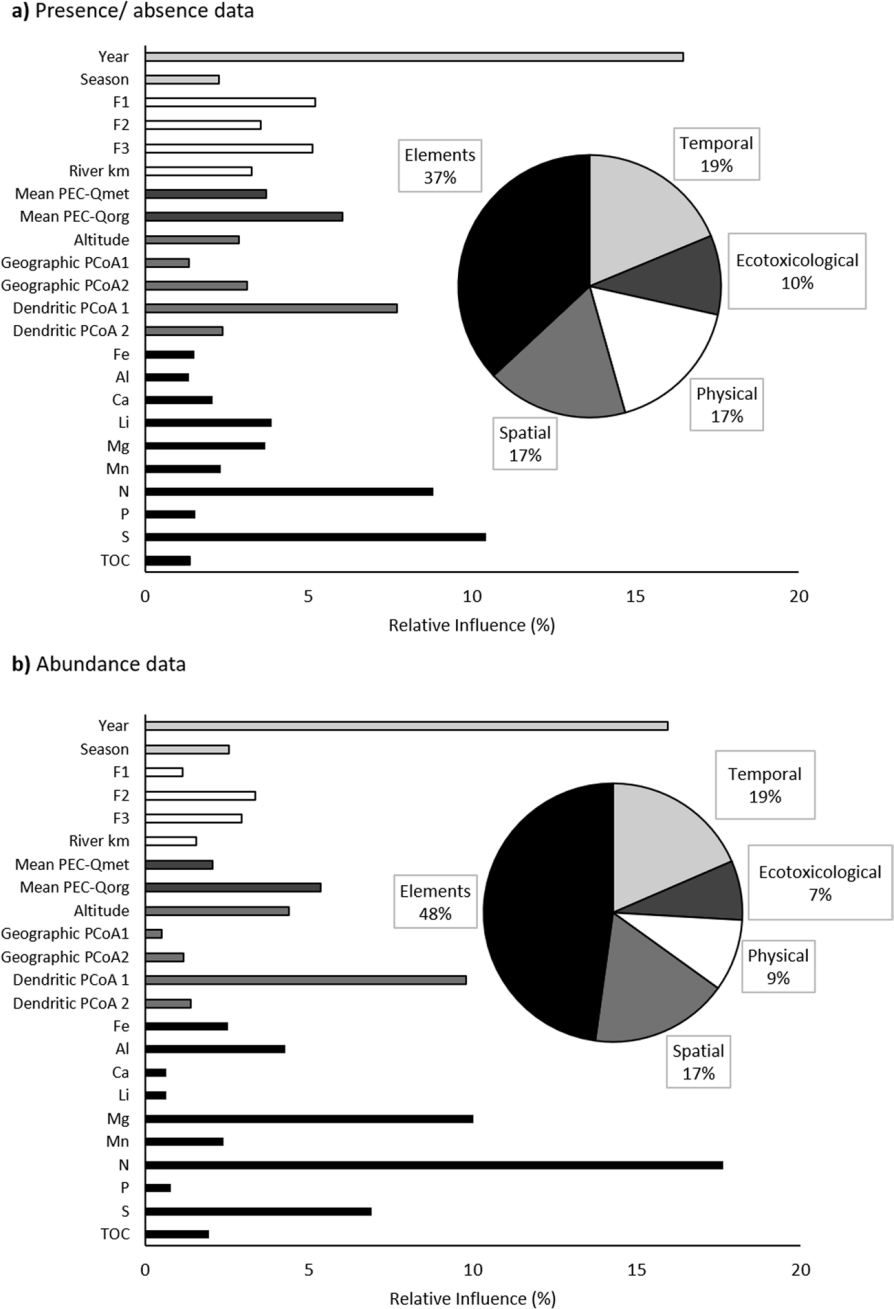
The relative influences of several predictors on nematode community similarities based on (**a**) presence/absence data and (**b**) abundance data determined using boosted regression tree models. The variables were assigned to five groups: Elements (black), temporal (light gray), spatial (middle gray), ecotoxicological (dark gray), and physical (white).

The RIs of the predictor variables of the models regarding the p/a and abundance data were similar (Fig. 3). In both cases, the parameter “elements” was the most important (p/a: 37%; abundance: 48%). Among the elements analyzed, N had the strongest association with community structure, as described by the abundance data (18%), followed by Mg (10%) and S (7%). Also, for the p/a data S (10%) and N (9%) were the most important elements. For both data types, the high RI values of the sampling year (16%) indicated a temporal dynamic for the nematode communities. Therefore, together with the predictor season the temporal parameter group was in both cases the second main source of variation. RI values of the spatial variables were 17% for both data types, with a high impact of the dendritic position of the sites (Dendritic PCoA 1: 8% and 10%). Furthermore, the community structure varied along physical gradients, especially the grain-size distribution (17% and 9%, respectively). The toxic potential of the sediment, indicated by the mean PEC-Q-values, had less influence (10% and 7%). Thus, overall, environmental variables accounted for 64% of the RIs and were therefore more important than spatial parameters in determining nematode community structure. The high RI value of the environmental parameter group was also influenced by the larger number of tested predictors. However, in a comparison restricted to single parameters, the RI values of the environmental predictors (N and S) were higher than those of the spatial ones, thus demonstrating that the number of predictors did not alter this conclusion.

A comparison of the results obtained with p/a vs. abundance data revealed a large shift in RI values: The impact of N as determined from the abundance data was twice as strong as that indicated by the species assemblages. Physical parameters and the ecotoxicological potential of the sites were, however, more important influences on the simple occurrence of a species than on its abundance.

### Predictions applied to the Rhine data

For the p/a data, both evaluation statistics showed significant correlations between the predicted and observed data for nematode community similarities in the Rhine samples with respect to the all NMDS axes. While the predictions were best for the second NMDS axis (correlation coefficients >0.75; p < 0.001), those for the first axis of community similarities were poorer but still satisfactory (r = 0.5 and ρ = 0.45; p < 0.001). By contrast, the overlap with observations was smallest for the NMDS axis 3 predictions (r = 0.28; ρ = 0.29 p < 0.03). Predictions calculated by the model trained with the first NMDS axis of the Elbe abundance data were not statistically validated (r = 0.10, p = 0.445; ρ = 0.12, p = 0.383) whereas for the second axis the predictions correlated well with the observations (r = 0.83; ρ = 0.77; p < 0.001). Predictions for the third axis were significantly associated with the real values but the correlation coefficients were small (r = 0.42, p < 0,001; ρ = 0.34, p = 0.008).

## Discussion

This is the first study to document the influence of environmental, spatial and temporal variables on the metacommunity structure of nematodes in a river network. As such, it contributes to closing the gap left by previous studies, in which meiobenthic organisms were rarely included. The calculated models identified the drivers of nematode community similarities in terms of species occurrence and abundances and yielded reliable predictions for community similarities in a distinct river system.

### Species sorting as a key driver of nematode metacommunity structure

Contrary to hypothesis (H1.1), our results showed that environmental rather than spatial variables were the prevailing influence on community similarities, thus suggesting that the communities were mostly controlled by species sorting. This implies nematode dispersal rates of intermediate strength^1,38^ and therefore that nematode species are sufficiently mobile to allow the colonization of favorable habitats, but their dispersal rates not high enough to induce strong mass (i.e. source-sink) effects. Our findings are in contrast with the results of the two other studies of meiobenthic metacommunities in stream networks^13,14^, in which ostracod community variances were better explained by spatial than by environmental predictors. Lower dispersal rates of ostracods than nematodes could explain why they are more strongly affected by dispersal limitation, although methodological differences in the respective studies could also have induced different outcomes, making such conclusions weak. Accordingly, the difference in spatial structure could have been an artifact of non-measured, spatially structured environmental variability^39^; alternatively, nonlinear environmental patterns may have been captures by spatial variables^40^. Further, both ostracod studies used p/a data, which mostly are less associated with species sorting than are abundance data^39^.

However, several other studies identified environmental conditions as key drivers of spatial community structure^41^, including that of diatoms^42^, macroinvertebrates^12,43–45^, and fishes^12,43,46^. The addition of nematodes to these already very different organismal groups provides further evidence of the high connectivity of sites within river networks, independent of the dispersal mode (passive or active) and aquatic zone (benthic or pelagic). Lentic systems, by contrast, are more isolated from surrounding aquatic habitats and may be more strongly influenced by spatial variables due to dispersal limitations^41,47^. Indeed, metacommunity studies of nematodes in lentic systems have revealed a significant, primary spatial structure of those communities, although these studies were conducted on spatial scales substantially smaller than the 480-km scale of the river network of the present study^48,49^.

Interestingly, a high RI value was assigned to the predictor year, showing a strong temporal dynamic within the nematode communities. In fact, with respect to species composition, the sampling year was more important than any of the other tested factors. Most metacommunity studies have not considered samples acquired over a timespan as long as that of our study, and the influence of temporal dynamics was for the most part assumed to be small^9^. By contrast, our study documented a shift in community assembly over time that may have been due to temporary changes in the environment, such as environmental disturbances, or to the incidental immigration and emigration of species. For example, nutrient addition, induced by the runoff of fertilizers from neighboring agricultural land, or extreme climate conditions, as occurred in the summer of 2003, could have led to strong changes in nematode community assemblies^50,51^.

### Shifts in the role of predictors for p/a vs abundance data

In terms of the abundance composition of species, the nitrogen content of the sediment was the strongest structuring gradient. This finding is in agreement with previous field and laboratory studies that identified the ability of nutrient enrichment to strongly shift the structure of nematode assemblages^50,51^. By contrast, nitrogen and other elements were poorer predictors for pure species assemblages, whereas the RI values of physical factors such as grain size distribution increased. Nematodes live in the interstitial, the size of which is dependent on the granulometry. Accordingly, nematode species of differing morphology (e.g. slender or compact) will depend on different sediment structures for growth and survival^52,53^. Thus, elements such as nitrogen may increase or decrease nematode abundances but are less important than physical factors regarding the basic occurrence of nematodes. Similarly, the ecotoxicological characteristics of the sediment seemed to be of greater importance for the simple presence rather than the abundance of a species.

### Watercourse distance is more important than Euclidean distance

Within the spatial variables, the RI values of the dendritic coordinates were higher than those of the geographic coordinates, which implied that, in structuring nematode communities, the watercourse distances of sites were more important than Euclidean, overland distances as it was assumed by hypothesis (H1.2).

The two processes that would lead to a spatial structure are dispersal limitation and mass effects. A detailed interpretation of the role played by those processes requires that the dispersal ability of the studied species be taken into account^42^, but for many aquatic organisms, including nematodes, this information is lacking^54^.

In many studies, the difference in the amount of variance explained by Euclidean and watercourse distances has been insignificant^3^. By contrast, in our study, watercourse position was almost twice as important for metacommunity structure as geographic position, indicating a difference between nematode dispersal ability overland vs. in water. Padial *et al*.^9^ compared the drivers of metacommunity structure with respect to a broad range of organismal groups and found that those with comparatively low dispersal ability, and therefore dependent on river network dispersal, are more strongly affected by watercourse than by Euclidean distances.

### Applicability of the model to the Rhine river system

Metacommunity studies in lotic systems have mainly focused on single river networks. However, of interest to environmental management is whether the detected patterns are applicable to other river networks. Studies that included more than one river system in their analysis have identified a river-specific influence on the diversity distribution^55,56^, although in this context the effect of river identity cannot be easily disentangled from that of its different spatial locations^8,57^.

In our study, the predicted values for the community similarity of nematodes based on p/a data correlated significantly with the real data but the predictions were less precise for the first and third NMDS axes. However, in both cases the values of the correlation coefficients obtained in the evaluation of the model were within the range of those of the cross-validation correlation coefficients (Tables S3, S4); specifically, the predictions within the Elbe data set were not better than those for the Rhine data set. Also, regarding the abundance data, the predictions of community similarities were best for the second axis whereas for the first and most important dimension the predictions did not reflect the observed values. This discrepancy can be explained by considering that the nitrogen content of the sediment, which was the main predictor in the Elbe model, did not cover such a wide gradient in the Rhine data (Table 1, Rhine: 1.4 ± 1.5 g/kg; Elbe: 3.2 ± 3.2 g/kg). Therefore, hypothesis (H2) could only partly be confirmed: Transfer of the model constructed based on the Elbe river system to the Rhine river system was effective only for p/a data. Yet, this result implied that similar processes shape the nematode species compositions of the two river networks.

## Conclusion

In addition to a high temporal dynamic of nematode communities, environmental predictors, especially the nitrogen content of the sediment, best explained the community composition of nematodes in terms of p/a and abundance. This result suggested that species sorting is the main driver structuring nematode metacommunities along the Elbe river network. Nevertheless, because shifts in the importance of predictors of community similarities with regard to both data types were identified, in future studies we recommend a stricter differentiation of the results according to the analyzed data. As most metacommunities have imprints of different mechanisms^41^, we also found indications that spatial factors, especially the river network position, influence nematode communities. Our model well predicted changes in the species composition in the Rhine river system but its predictive ability regarding abundance compositions was limited. However, our study shows that the consequences of changes in environmental factors or habitat connectivity for aquatic communities, irrespective of river network identity, can be quantified.

## Supplementary information

Supplementary Information

Table S1

## Data Availability

All data are included as Supplementary Information.
