# Supplementary material for: Environmental factors and river network position allow prediction of benthic community assemblies: A model of nematode metacommunities
Source: Sci Rep. 2019 Oct 11;9:14716. doi: 10.1038/s41598-019-51245-2 (PMC6789110; doi:10.1038/s41598-019-51245-2)
Supplement: Supplementary file 1 — Supplementary Information [file 41598_2019_51245_MOESM1_ESM.docx]

**Supplementary Information for**

Environmental factors and river network position allow prediction of benthic community assemblies: A model of nematode metacommunities

Birgit Gansfort & Walter Traunspurger

Corresponding author: Birgit Gansfort

Email: birgit.gansfort@uni-bielefeld.de

**This PDF file includes:**

Supplementary text

Figure S1

Tables S2 to S4

Table S1 is supplied separately (spreadsheet)

Supplementary Information Text

**Calculation of the weighted average for the relative influence (RI) values.**

Example of presence/absence data, for stress reduction (SR); 100% was defiined as *1-stress_dim3_ =1-0.19=0.81*

| Dimension | Stress | SR |
| --- | --- | --- |
| 0 | 1.00 |  |
| 1 | 0.41 | 0.73 |
| 2 | 0.26 | 0.19 |
| 3 | 0.19 | 0.08 |

Calculation of the weighted average of the RI values:

$$RI_{mean}=\sum_{i=1}^{5} RI_{NMDSi}*SR_{dimi}$$

a)


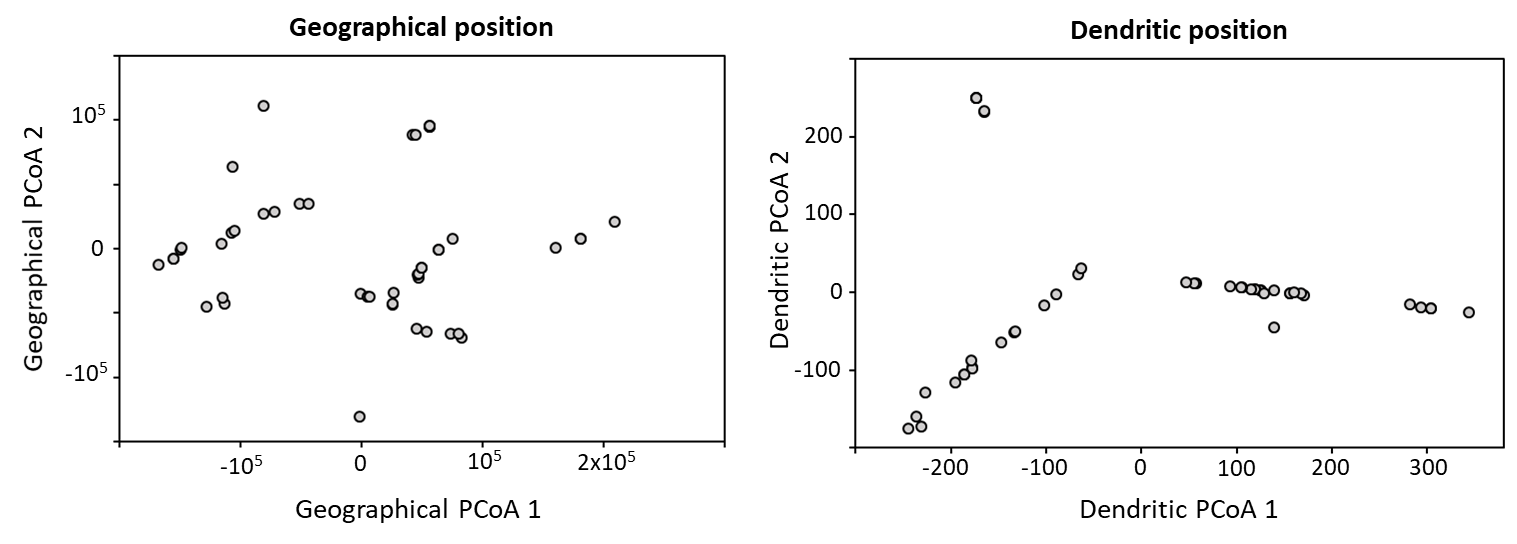


**Figure S1**: Principal coordinates analysis (PCoA) plots of the sampling sites within the Elbe (a) and Rhine (b) river networks, according to geographical position (left), based on the geodesic distances of the sites, and their dendritic position (right), based on the distances of sites along the watercourse


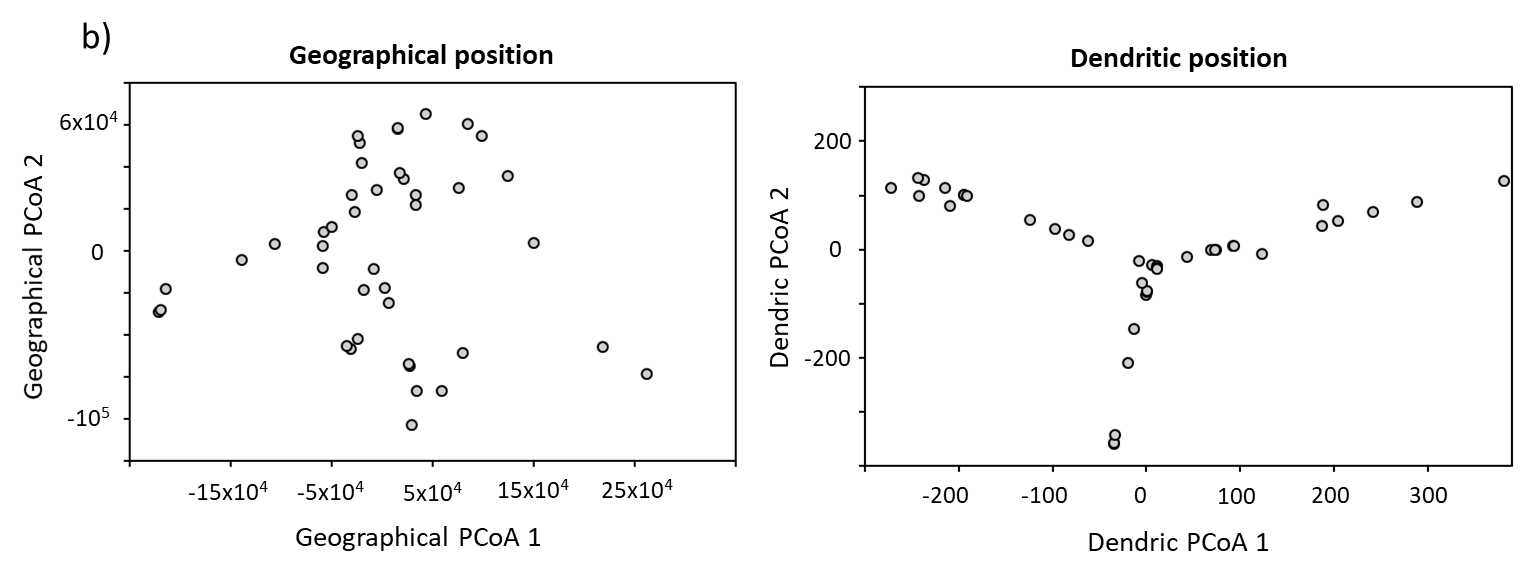


**Figure S1** *continued*

Table S2: List of nematode species and numbers of their presences in 115 samples of Elbe and 59 samples of Rhine river system

| **Species** | **Number of presences in** | | |
| --- | --- | --- | --- |
|  | **Total** | **Rhine** | **Elbe** |
| *Achromadora longicauda* Schneider, 1937 | 4 | 3 | 1 |
| *Achromadora micoletzkyi* (Stefanski, 195) | 16 | 10 | 6 |
| *Achromadora ruricola* (de Man, 188) | 19 | 8 | 11 |
| *Achromadora* spec. | 3 | 0 | 3 |
| *Achromadora tenax* (de Man, 1876) | 8 | 3 | 5 |
| *Achromadora terricola* (de Man, 188) | 3 | 2 | 1 |
| *Acrobeles ciliatus* Linstow, 1877 | 1 | 0 | 1 |
| *Acrobeloides buetschlii* (de Man, 884) | 3 | 0 | 3 |
| *Aglenchus agricola* (de Man, 1884) | 3 | 2 | 1 |
| *Aglenchus* spec. | 1 | 1 | 0 |
| *Alaimus meyli* Andrássy, 1961 | 5 | 3 | 2 |
| *Alaimus parvus* Thorne, 1939 | 15 | 11 | 4 |
| *Alaimus primitivus* de Man, 1880 | 13 | 11 | 2 |
| *Alaimus proximus* Thorn, 1939 | 5 | 3 | 2 |
| *Alaimus* spec. 1 | 2 | 1 | 1 |
| *Alaimus* spec. 2 | 3 | 1 | 2 |
| *Amphidelus* cf. *elegans* (de Man, 1921) | 5 | 2 | 3 |
| *Amplimerlinius* spec. | 1 | 0 | 1 |
| *Anaplectus granulosus* (Bastian, 1865) | 11 | 3 | 8 |
| *Anatonchus dolichurus* (Ditlevsen, 1911) | 1 | 1 | 0 |
| *Anatonchus tridentatus* (de Man, 1876) | 1 | 1 | 0 |
| *Aphanolaimus aquaticus* Daday, 1894 | 36 | 18 | 18 |
| *Aphanolaimus attentus* de Man, 1880 | 2 | 2 | 0 |
| *Aphelenchoides bicaudatus* (Imamura, 1931) | 2 | 0 | 2 |
| *Aphelenchoides fluviatilis* Andrássy, 1960 | 4 | 3 | 1 |
| *Aphelenchoides fragariae* (Ritzema Bos, 1890) | 1 | 1 | 0 |
| *Aphelenchoides parietinus* (Bastian, 1865) | 4 | 3 | 1 |
| *Aphelenchoides* spec. | 26 | 4 | 22 |
| *Aphelenchoides subparietinus* Sanwal, 1961 | 1 | 1 | 0 |
| *Aphelenchus avanae* Bastian, 1865 | 1 | 1 | 0 |
| *Aphelenchus* spec. | 3 | 3 | 0 |
| *Aporcelaimellus obtusicaudatus* (Bastian, 1865) | 17 | 9 | 8 |
| *Aporcelaimellus* spec. | 13 | 3 | 10 |
| *Aporcelaimus superbus* (de Man, 1880) | 1 | 1 | 0 |
| *Aulolaimoides* cf. *phoxodorus* Andrássy, 1964 | 2 | 2 | 0 |
| *Bastiania gracilis* de Man, 1876 | 3 | 2 | 1 |
| *Bastiania longicaudata* de Man, 1880 | 1 | 1 | 0 |
| *Bastiania* spec. | 2 | 0 | 2 |
| *Bastiania uncinata* Andrássy, 1991 | 1 | 1 | 0 |
| *Bitylenchus* cf. *bryobius* (Sturhan, 1966) | 2 | 1 | 1 |
| *Bitylenchus dubius* (Bütschli, 1873) | 8 | 5 | 3 |
| *Brevitobrilus* spec. | 1 | 0 | 1 |
| *Brevitobrilus stefanskii* (Micoletzky, 1925) | 93 | 23 | 70 |
| *Bursilla monhystera* (Buetschli, 1873) | 14 | 5 | 9 |
| *Cephalenchus* spec. | 3 | 2 | 1 |
| Cephalobidae unidentified | 5 | 3 | 2 |
| *Cephalobus persegnis* Bastian, 1865 | 47 | 22 | 25 |
| *Chiloplectus andrassyi (Timm, 1971)* | 1 | 1 | 0 |
| *Chromadoridae* gen. spec. 1 | 7 | 2 | 5 |
| *Chromadoridae* gen. spec. 2 | 1 | 1 | 0 |
| *Chromadorina astacicola* (Schneider, 1932) | 1 | 1 | 0 |
| *Chromadorina bercziki* Andrássy, 1962 | 6 | 4 | 2 |
| *Chromadorina bioculata* (Schulze in Carus, 1857) | 55 | 19 | 36 |
| *Chromadorina* spec. | 2 | 0 | 2 |
| *Chromadorina viridis* (Linstow, 1876) | 15 | 6 | 9 |
| *Chromadorita leuckarti* (de Man, 1876) | 61 | 15 | 46 |
| *Chronogaster* cf. *lissa* Loof, 1973 | 1 | 1 | 0 |
| *Chronogaster* spec. | 1 | 0 | 1 |
| *Chronogaster typica* (de Man, 1921) | 1 | 0 | 1 |
| *Clarkus papillatus* (Bastian, 1865) | 1 | 1 | 0 |
| *Clavicaudoides* cf. *clavicaudatus* (Altherr, 1953) | 1 | 1 | 0 |
| *Coslenchus costatus* (de Man, 1921) | 16 | 7 | 9 |
| *Crassolabium circuliferum* (Loof, 1961) | 1 | 1 | 0 |
| *Crassolabium ettersbergensis* (de Man, 1885) | 3 | 2 | 1 |
| *Criconema annuliferum* (de Man, 1921) | 2 | 1 | 1 |
| *Criconema demani* Micoletzky, 1925 | 3 | 3 | 0 |
| *Criconematidae* gen. spec. | 2 | 1 | 1 |
| *Criconemoides informis (*Micoletzky, 1922) | 1 | 0 | 1 |
| *Criconemoides morgensis* (Hofmänner in Hofmänner & Menzel, 1914) | 5 | 2 | 3 |
| *Criconemoides* spec. | 6 | 4 | 2 |
| *Crocodorylaimus flavomaculatus* (Linstow, 1876) | 2 | 1 | 1 |
| *Cruznema tripartitum* (Linstow, 1906) | 2 | 0 | 2 |
| *Cryptonchus tristis* (Ditlevsen, 1911) | 1 | 0 | 1 |
| *Curviditis curvicaudata* (Schneider, 1866) | 1 | 0 | 1 |
| *Cuticularia oxycerca* (de Man, 1895) | 12 | 10 | 2 |
| *Cuticularia* spec. | 6 | 0 | 6 |
| *Cylindrolaimus communis* de Man, 1880 | 12 | 8 | 4 |
| *Cylindrotheristus vesentinae* (Andrássy, 1962) | 3 | 0 | 3 |
| *Daptonema dubium* Bütschli, 1873 | 78 | 35 | 43 |
| *Diphterophora communis* de Man, 1880 | 2 | 2 | 0 |
| *Diplogaster rivalis* (Leydig, 1854) | 5 | 2 | 3 |
| *Diplogaster* spec. 1 | 7 | 2 | 5 |
| *Diplogaster* spec. 2 | 1 | 1 | 0 |
| *Diplogasteridae* gen. spec. 1 | 17 | 7 | 10 |
| *Diplogasteridae* gen. spec. 2 | 3 | 0 | 3 |
| *Diplogasteritus consobrinus* (de Man, 1920) | 19 | 11 | 8 |
| *Diplogasteritus nudicapitatus* (Steiner, 1914) | 1 | 1 | 0 |
| *Diploscapter coronatus* (Cobb, 1893) | 28 | 9 | 19 |
| *Ditylenchus intermedius* (de Man, 1880) | 1 | 0 | 1 |
| *Dorylaimidae* gen. spec. 1 | 4 | 0 | 4 |
| *Dorylaimidae* gen. spec. 2 | 1 | 0 | 1 |
| *Dorylaimidae* gen. spec. 3 | 7 | 0 | 7 |
| *Dorylaimidae* gen. spec. 4 | 1 | 0 | 1 |
| *Dorylaimoides limnophilus* (de Man, 1880) | 3 | 2 | 1 |
| *Dorylaimus* spec. 1 | 6 | 0 | 6 |
| *Dorylaimus* spec. 2 | 1 | 0 | 1 |
| *Dorylaimus* spec. 3 | 1 | 0 | 1 |
| *Dorylaimus stagnalis* Dujardin, 1845 | 113 | 45 | 68 |
| *Doryllium* cf*. zeelandium* (de Man, 1876) | 1 | 1 | 0 |
| *Doryllium minus* Jairajpuri, 1963 | 1 | 1 | 0 |
| *Epidorylaimus agilis* (de Man, 1880) | 12 | 9 | 3 |
| *Epidorylaimus* spec. | 11 | 3 | 8 |
| *Epitobrilus medius* (Schneider, 1916) | 4 | 0 | 4 |
| *Ethmolaimus pratensis* de Man, 1880 | 24 | 14 | 10 |
| *Ethmolaimus* spec. | 1 | 0 | 1 |
| *Eucephalobus mucronatus* (Kozlowska & Roguska-Wasilewska, 1963) | 12 | 6 | 6 |
| *Eucephalobus oxyuroides* (de Man, 1876) | 5 | 2 | 3 |
| *Eucephalobus* spec. | 1 | 1 | 0 |
| *Eucephalobus striatus* (Bastian, 1865) | 3 | 2 | 1 |
| *Eudorylaimus acuticauda* (de Man, 180) | 10 | 6 | 4 |
| *Eudorylaimus carteri* (Bastian, 1865) | 10 | 5 | 5 |
| *Eudorylaimus centrocercus* (de Man, 1880) | 10 | 3 | 7 |
| *Eudorylaimus* cf. *brevis* (Altherr, 1952) | 1 | 0 | 1 |
| *Eudorylaimus* cf. *consobrinus* (De Man 1917) | 1 | 1 | 0 |
| *Eumonhystera andrassyi* (Biro, 1969) | 1 | 1 | 0 |
| *Eumonhystera barbata* Andrássy, 1981 | 32 | 19 | 13 |
| *Eumonhystera dispar* (Bastian, 1865) | 63 | 20 | 43 |
| *Eumonhystera filiformis* (Bastian, 1865) | 167 | 54 | 113 |
| *Eumonhystera longicaudatula* (Gerlach & Riemann, 1973) | 76 | 32 | 44 |
| *Eumonhystera pseudobulbosa* (Daday, 1896) | 111 | 40 | 71 |
| *Eumonhystera similis* (Bütschli, 1873) | 5 | 1 | 4 |
| *Eumonhystera simplex* (de Man, 1880) | 45 | 20 | 25 |
| *Eumonhystera* spec. 1 | 1 | 0 | 1 |
| *Eumonhystera* spec. 2 | 19 | 8 | 11 |
| *Eumonhystera* spec. 3 | 5 | 1 | 4 |
| *Eumonhystera* spec. 4 | 4 | 1 | 3 |
| *Eumonhystera vulgaris* (de Man, 1880) | 81 | 35 | 46 |
| *Euteratocephalus palustris* (de Man, 1880) | 4 | 2 | 2 |
| *Fictor fictor* (Bastian, 1865) | 2 | 0 | 2 |
| *Filenchus thornei* (Andrássy, 1954) | 1 | 1 | 0 |
| *Filenchus vulgaris* (Brzeski, 1963) | 69 | 35 | 34 |
| *Geomonhystera villosa* (Bütschli, 1873) | 1 | 0 | 1 |
| *Helicotylenchus pseudorobustus* (Steiner, 1914) | 10 | 6 | 4 |
| *Helicotylenchus* spec. 1 | 21 | 11 | 10 |
| *Helicotylenchus* spec. 2 | 1 | 0 | 1 |
| *Helicotylenchus* spec. 3 | 8 | 5 | 3 |
| *Helicotylenchus varicaudatus* Yuen, 1964 | 4 | 3 | 1 |
| *Hemicycliophora* spec. 1 | 5 | 2 | 3 |
| *Hemicycliophora thienemanni* (Schneider, 1925) | 1 | 1 | 0 |
| *Hemicycliophora thornei* Goodey, 1963 | 1 | 1 | 0 |
| *Hemicycliophora typica* de Man, 1921 | 5 | 2 | 3 |
| *Heterocephalobus elongatus* (de Man, 1880) | 10 | 6 | 4 |
| *Heterocephalobus* spec. | 8 | 3 | 5 |
| *Hirschmaniella gracilis* (de Man, 1880) | 10 | 6 | 4 |
| *Ironus* cf. *macramphis* Stekhoven & Teunissen, 1938 | 1 | 0 | 1 |
| *Ironus ignavus* Bastian, 1865 | 7 | 6 | 1 |
| *Ironus longicaudatus* de Man, 1884 | 1 | 0 | 1 |
| *Ironus* spec. | 1 | 0 | 1 |
| *Ironus tenuicaudatus* de Man, 1876 | 30 | 14 | 16 |
| *Laimydorus* cf. *pseudostagnalis* (Micoletzky, 1927) | 2 | 1 | 1 |
| *Lelenchus leptosoma* (de Man, 1880) | 5 | 4 | 1 |
| *Longidorella* spec. | 1 | 0 | 1 |
| *Malenchus byrophilus* (Steiner, 1914) | 1 | 1 | 0 |
| *Malenchus* spec. | 1 | 1 | 0 |
| *Merlinius brevidens* (Allen, 1955) | 6 | 1 | 5 |
| Mermithidae | 12 | 5 | 7 |
| *Mesocriconema rusticum* (Micoletzky, 1915) | 5 | 4 | 1 |
| *Mesodoryaimus* cf. *conurus* (Thorne, 1939) | 9 | 8 | 1 |
| *Mesodorylaimus bastiani* (Bütschli, 1873) | 25 | 13 | 12 |
| *Mesodorylaimus paetzoldi* Altherr, 1965 | 3 | 3 | 0 |
| *Mesodorylaimus rhenanus* Altherr, 1965 | 2 | 0 | 2 |
| *Mesodorylaimus* spec. 1 | 3 | 0 | 3 |
| *Mesodorylaimus* spec. 2 | 62 | 20 | 42 |
| *Mesodorylaimus subtiliformis* (Andrássy, 1959) | 5 | 2 | 3 |
| *Mesorhabditis spiculigera* (Steiner, 1936) | 4 | 1 | 3 |
| *Metateratocephalus crassidens* (de Man, 1880) | 1 | 0 | 1 |
| *Metateratocephalus* spec. | 2 | 0 | 2 |
| *Miculenchus salvus* Andrássy, 1959 | 1 | 0 | 1 |
| *Monhystera* cf*. stagnalis/paladicola* | 15 | 7 | 8 |
| *Monhystera lemani* Juget, 1969 | 1 | 1 | 0 |
| *Monhystera paludicola* de Man, 1880 | 134 | 45 | 89 |
| *Monhystera* spec. 1 | 2 | 2 | 0 |
| *Monhystera* spec. 2 | 9 | 4 | 5 |
| *Monhystera* spec. 3 | 3 | 0 | 3 |
| *Monhystera* spec. 4 | 1 | 0 | 1 |
| *Monhystera* spec. 5 | 1 | 0 | 1 |
| *Monhystera stagnalis* Bastian, 1865 | 100 | 41 | 59 |
| *Monhystrella macrura* (de Man, 1880) | 4 | 1 | 3 |
| *Monhystrella paramacrura* (Meyl, 1953) | 38 | 19 | 19 |
| *Monhystrella thermophila* (Meyl, 1953) | 2 | 1 | 1 |
| *Mononchus aquaticus* Coetzee, 1968 | 54 | 18 | 36 |
| *Mononchus niddensis* Skwarra, 1921 | 2 | 0 | 2 |
| *Mononchus pulcher* Andrássy, 1993 | 1 | 0 | 1 |
| *Mononchus* spec. | 1 | 0 | 1 |
| *Mononchus truncatus* Bastian, 1865 | 34 | 20 | 14 |
| *Mononchus tunbridgensis* Bastian, 1865 | 39 | 11 | 28 |
| *Mylonchulus brachyuris* (Bütschli, 1873) | 2 | 1 | 1 |
| *Mylonchulus sigmaturus* Cobb, 1917 | 1 | 1 | 0 |
| *Mylonchulus* spec. | 1 | 0 | 1 |
| *Neodolichorhynchus lamelliferus* (de Man, 1880) | 2 | 1 | 1 |
| *Neodolichorhynchus* spec. | 1 | 1 | 0 |
| *Neotobrilus longus* (Leidy, 1852) | 16 | 9 | 7 |
| *Nygolaimus brachyuris* (de Man, 1880) | 1 | 1 | 0 |
| *Nygolaimus* spec. | 1 | 1 | 0 |
| *Odontolaimus chlorurus* de Man, 1880 | 1 | 0 | 1 |
| *Ogma* cf. *danubialis* Andrássy, 1985 | 1 | 1 | 0 |
| *Ogma* cf*. octangularis* (Cobb, 1914) | 1 | 0 | 1 |
| *Opisthodorylaimus sylphoides* (Williams, 1959) | 1 | 1 | 0 |
| *Panagrolaimidae* gen. spec. | 2 | 1 | 1 |
| *Panagrolaimus rigidus* (Schneider, 1866) | 2 | 0 | 2 |
| *Panagrolaimus* spec. 1 | 31 | 15 | 16 |
| *Panagrolaimus* spec. 2 | 1 | 0 | 1 |
| *Panagrolaimus* spec. 3 | 1 | 0 | 1 |
| *Paractinolaimus macrolaimus* (de Man, 1880) | 5 | 2 | 3 |
| *Paramphidelus* cf. *paramonovi* (Eliashvili, 1971) | 1 | 1 | 0 |
| *Paramphidelus dolichurus* (de Man, 1876) | 5 | 4 | 1 |
| *Paramphidelus uniformis* (Thorne, 1939) | 1 | 1 | 0 |
| *Paraphanolaimus anisitsi* (Daday, 1905) | 2 | 2 | 0 |
| *Paraphanolaimus behningi* Micoletzky, 1923 | 4 | 3 | 1 |
| *Paraplectonema pedunculatum* (Hofmänner, 1913) | 30 | 28 | 2 |
| *Paratylenchus* cf*. microdorus Andrássy, 1959* | 1 | 0 | 1 |
| *Paratylenchus nanus* Cobb, 1923 | 1 | 0 | 1 |
| *Paroigolaimella bernensis* (Steiner, 1914) | 3 | 0 | 3 |
| *Pellioditis pellioides* (Bütschli, 1873) | 1 | 0 | 1 |
| *Plectus aquatilis* Andrássy, 1985 | 45 | 28 | 17 |
| *Plectus cirratus* Bastian, 1865 | 20 | 12 | 8 |
| *Plectus geophilus* de Man, 1880 | 5 | 2 | 3 |
| *Plectus longicaudatus* Bütschli, 1873 | 3 | 1 | 2 |
| *Plectus opisthocirculus* Andrássy, 1952 | 21 | 9 | 12 |
| *Plectus parietinus* Bastian, 1865 | 1 | 0 | 1 |
| *Plectus parvus* Bastian, 1865 | 9 | 4 | 5 |
| *Plectus rhizophilus* de Man, 1880 | 4 | 2 | 2 |
| *Plectus* spec. 1 | 2 | 0 | 2 |
| *Plectus* spec. 2 | 1 | 0 | 1 |
| *Plectus* spec. 3 | 19 | 5 | 14 |
| *Plectus* spec. 4 | 1 | 1 | 0 |
| *Plectus tenuis* Bastian, 1865 | 1 | 1 | 0 |
| *Pratylenchoides* cf*. crenicauda* (Winslow, 1958) | 3 | 1 | 2 |
| *Pratylenchoides* cf. *riparius* (Andrássy, 1985) | 1 | 0 | 1 |
| *Pratylenchus pratensis* (de Man, 1880) | 2 | 2 | 0 |
| *Pratylenchus* spec. | 6 | 3 | 3 |
| *Prismatolaimus* cf. *dolichurus* de Man, 1880 | 3 | 3 | 0 |
| *Prismatolaimus* cf. *tenuicaudatus* Schuurmans Stekhoven, 1951 | 5 | 5 | 0 |
| *Prismatolaimus intermedius* (Bütschli, 1873) | 47 | 30 | 17 |
| *Prismatolaimus* spec. 1 | 2 | 0 | 2 |
| *Prismatolaimus* spec. 2 | 1 | 1 | 0 |
| *Prodesmodora arctica* (Mulvey, 1969) | 3 | 0 | 3 |
| *Prodesmodora circulata* (Micoletzky, 1913) | 11 | 4 | 7 |
| *Prodesmodora terricola* Altherr, 1952 | 1 | 0 | 1 |
| *Prodorylaimus brigdammensis* (de Man 1876) | 5 | 5 | 0 |
| *Prodorylaimus* cf. *longicaudatoides* Altherr, 1968 | 1 | 1 | 0 |
| *Prodorylaimus rotundiceps* Loof, 1985 | 1 | 0 | 1 |
| *Prodorylaimus* spec. | 11 | 9 | 2 |
| *Propanogrolaimus* cf*. thienemanni* (de Man, 1880) | 1 | 0 | 1 |
| *Pseudoaulolaimus anchilocaudatus* Imamura, 1931 | 1 | 1 | 0 |
| *Psilenchus aestuarius* Andrássy, 1962 | 2 | 1 | 1 |
| *Punctodora dudichi* Andrassy, 1966 | 2 | 1 | 1 |
| *Punctodora ratzeburgensis* (Linstow, 1876) | 9 | 6 | 3 |
| *Rhabditidae* gen spec. 1 | 41 | 15 | 26 |
| *Rhabditidae* gen spec. 2 | 2 | 2 | 0 |
| *Rhabditidae* gen spec. 3 | 7 | 6 | 1 |
| *Rhabditidae* gen spec. 4 | 1 | 0 | 1 |
| *Rhabditis gracilicauda* de Man, 1876 | 3 | 1 | 2 |
| *Rhabditis* spec. 1 | 1 | 1 | 0 |
| *Rhabditis* spec. 2 | 4 | 2 | 2 |
| *Rhabdolaimus aquaticus* de Man, 1880 | 3 | 0 | 3 |
| *Rhabdolaimus* cf. *limnophilus (Soos, 1937)* | 1 | 0 | 1 |
| *Rhabdolaimus terrestris* de Man, 1880 | 6 | 4 | 2 |
| *Rhomborhabditis teres* (Schneider, 1866) | 5 | 3 | 2 |
| *Rotylenchus* cf. *goodeyi* Loof & Oostenbrink, 1958 | 1 | 0 | 1 |
| *Rotylenchus robustus* (de Man, 1876) | 2 | 2 | 0 |
| *Rotylenchus* spec. | 2 | 2 | 0 |
| *Scleramphidelus lemani* (Stefanski, 1914) | 4 | 3 | 1 |
| *Semitobrilus pellucidus* (Bastian, 1865) | 64 | 26 | 38 |
| Species 1 | 1 | 0 | 1 |
| Species 2 | 1 | 0 | 1 |
| Species 3 | 2 | 1 | 1 |
| Species 4 | 3 | 1 | 2 |
| Species 5 | 1 | 0 | 1 |
| Species 6 | 1 | 0 | 1 |
| Species 7 | 1 | 0 | 1 |
| Species 8 | 2 | 2 | 0 |
| *Teratocephalus costatus* Andrássy, 1958 | 3 | 2 | 1 |
| *Teratocephalus tenuis* Andrássy, 1956 | 1 | 1 | 0 |
| *Theristus agilis* (de Man, 1880) | 11 | 6 | 5 |
| *Theristus* spec. 1 | 1 | 0 | 1 |
| *Theristus* spec. 2 | 1 | 0 | 1 |
| *Thonus* spec. | 3 | 2 | 1 |
| *Thornia propinqua* (Paesler, 1941) | 8 | 6 | 2 |
| *Thornia* spec. | 9 | 5 | 4 |
| *Tobrilidae* gen. sp. | 4 | 1 | 3 |
| *Tobrilus diversipapillatus* Daday, 1905 | 23 | 3 | 20 |
| *Tobrilus gracilis* Bastian, 1865 | 149 | 52 | 97 |
| *Tobrilus* spec. 1 | 9 | 2 | 7 |
| *Tobrilus* spec. 2 | 2 | 0 | 2 |
| *Tobrilus* spec. 3 | 1 | 0 | 1 |
| *Tobrilus* spec. 4 | 1 | 0 | 1 |
| *Tobrilus* spec. 5 | 9 | 9 | 0 |
| *Trichodorus primitivus* (de Man, 1880) | 1 | 1 | 0 |
| *Tripyla affinis* de Man, 1880 | 3 | 2 | 1 |
| *Tripyla filicaudata* de Man, 1880 | 11 | 2 | 9 |
| *Tripyla glomerans* Bastian, 1865 | 72 | 16 | 56 |
| *Tripyla setifera* Bütschli, 1873 | 3 | 1 | 2 |
| *Trischistoma arenicola* (de Man, 1880) | 9 | 9 | 0 |
| *Trischistoma monohystera* (de Man, 1880) | 2 | 2 | 0 |
| *Tylenchidae* gen. spec. 1 | 6 | 3 | 3 |
| *Tylenchidae* gen. spec. 10 | 4 | 3 | 1 |
| *Tylenchidae* gen. spec. 11 | 1 | 1 | 0 |
| *Tylenchidae* gen. spec. 12 | 2 | 2 | 0 |
| *Tylenchidae* gen. spec. 2 | 1 | 0 | 1 |
| *Tylenchidae* gen. spec. 3 | 1 | 0 | 1 |
| *Tylenchidae* gen. spec. 4 | 11 | 4 | 7 |
| *Tylenchidae* gen. spec. 5 | 1 | 1 | 0 |
| *Tylenchidae* gen. spec. 6 | 5 | 0 | 5 |
| *Tylenchidae* gen. spec. 7 | 3 | 3 | 0 |
| *Tylenchidae* gen. spec. 8 | 38 | 14 | 24 |
| *Tylenchidae* gen. spec. 9 | 5 | 3 | 2 |
| *Tylencholaimus minimus* de Man, 1876 | 3 | 3 | 0 |
| *Tylenchus davainei* Bastian, 1865 | 29 | 16 | 13 |
| *Tylenchus* spec*.* 1 | 25 | 12 | 13 |
| *Tylenchus* spec. 2 | 2 | 1 | 1 |
| *Tylenchus* spec. 3 | 7 | 2 | 5 |
| *Tylenchus* spec. 4 | 8 | 7 | 1 |
| *Wilsonema otophorum* (de Man, 1880) | 2 | 1 | 1 |

**Table S3:** Statistics of the boosted regression tree (BRT) models for nematode communities (based on presence/absence and abundance data). For each response variable, the final number of trees. the model’s deviance, and the cross-validation (CV) statistics are shown.

| **Response variable** | **No. of trees** | **Mean total deviance** | **Mean residual deviance** | **Estimated CV deviance** | **Training data correlation** | **CV correlation** | |
| --- | --- | --- | --- | --- | --- | --- | --- |
| **Presence/ absence data** | | | | | | |  |
| NMDS axis 1 | 5300 | 0.14 | 0.53 | 0.12 | 0.86 | 0.23 | |
| NMDS axis 2 | 2800 | 0.12 | 0.03 | 0.05 | 0.88 | 0.79 | |
| NMDS axis 3 | 4550 | 0.07 | 0.03 | 0.06 | 0.85 | 0.48 | |
| **Abundance data** |  |  |  |  |  |  | |
| NMDS axis 1 | 1950 | 0.23 | 0.14 | 0.22 | 0.74 | 0.22 | |
| NMDS axis 2 | 3050 | 0.16 | 0.03 | 0.05 | 0.92 | 0.83 | |
| NMDS axis 3 | 2900 | 0.13 | 0.04 | 0.08 | 0.84 | 0.69 | |

**Table S4**: Correlation and linear regression of the predicted and observed values for nematode community similarities (abundance) and species assemblages (presence/absence) at 59 sampling sites along the Rhine river system

| **Statistic** | **NMDS1** | **NMDS2** | **NMDS3** |
| --- | --- | --- | --- |
| **Presence/ Absence data** | | | |
| **Pearson** |  |  |  |
| r | **0.50** | **0.77** | **0.28** |
| p | **<0.001** | **<0.001** | **0.029** |
| **Spearman** |  |  |  |
| rho | **0.45** | **0.75** | **0.29** |
| p | **<0.001** | **<0.001** | **0.028** |
| **Abundance data** | | | |
| **Pearson** |  |  |  |
| r | 0.10 | **0.83** | **0.42** |
| p | 0.445 | **<0.001** | **0.001** |
| **Spearman** |  |  |  |
| rho | 0.12 | **0.77** | **0.34** |
| p | 0.383 | **<0.001** | **0.008** |
